# Perceived parental warmth attenuates the link between perceived parental rejection and rumination in Chinese early adolescents: two conditional moderation models

**DOI:** 10.3389/fpsyt.2024.1294291

**Published:** 2024-01-24

**Authors:** Fanfei Meng, Cuiping Cheng, Yuntian Xie, Haihua Ying, Xinling Cui

**Affiliations:** ^1^ School of Education Science, Nanjing Normal University, Nanjing, China; ^2^ School of Preschool Education, Changsha Normal University, Changsha, China; ^3^ School of Teacher Development, Chongqing University of Education, Chongqing, China; ^4^ International School, Hohai University, Nanjing, China; ^5^ School of Education Science, Northwest Normal University, Lanzhou, China

**Keywords:** Chinese early adolescent, parental rejection, parental warmth, rumination, conditional moderation

## Abstract

**Background:**

Prior studies have explored the association between perceived parental rejection-warmth and adolescents’ rumination, but it is unclear whether the interaction between perceived parental rejection and warmth can predict adolescents’ rumination in a Chinese context and whether this interaction varies by children’s gender during the post-COVID-19 era.

**Objective:**

This study aimed to address these issues in Chinese early adolescents from a family system perspective.

**Methods:**

A total of 910 adolescents (*M*_age_ = 13.63, 48.50% female) from two middle schools in Chongqing and Changsha, China participated in the survey, answering measures for demographics, perceived parental rejection-warmth, and rumination.

**Results:**

The results show that adolescents’ rumination was positively related to perceived paternal rejection (*r* = 0.326, *p* <.001) and maternal rejection (*r* = 0.343, *p* <.001), and negatively related to perceived paternal warmth (*r* = -.184, *p* <.001) and maternal warmth (*r* = -0.125, *p* <.001). Moreover, perceived maternal warmth significantly moderated the link between perceived paternal rejection and adolescents’ rumination (boot effect = -0.066, 95CI% = [-0.124, -0.010]), but this moderating effect is only presented in boys not in girls (boot effect = -0.063, 95CI% = [-0.015, 0.140]). However, perceived paternal warmth moderated the link between perceived maternal rejection and rumination in adolescents (boot effect = -0.052, 95CI% = [-0.103, -0.001]), and this interaction varied by adolescents’ gender (boot effect = 0.103, 95CI% = [0.029, 0.177]).

**Conclusions:**

Perceived Parental rejection and parental warmth co-exist in the Chinese family system, and they exert an interactive effect on adolescents’ rumination depending on their gender. It implies that both parents should be more accepting, caring, considerate, and warm toward their daughters, and it is also in line with the tradition and status quo of parenting in Chinese families. These findings have implications for Chinese parental co-parenting practices in families with adolescents and adolescence mental health counseling work.

## 1 Introduction

The COVID-19 pandemic has negatively impacted adolescents’ mental health, leading to symptoms such as depression, anxiety, and rumination (1, 2). Rumination is a mode of responding to distress that involves repetitively and passively focusing on symptoms of distress and the possible causes and consequences of those symptoms (3). It tends to appear in early adolescence when individuals try to deal with stressful life events (4–6). People who are ruminating remain fixated on their problems and their feelings about them rather than taking action to solve them (3). For adolescents, rumination was found to be a vital indicator of many negative physical and mental consequences, such as depression, anxiety (7, 8), and self-injury (9), as well as other internalizing and externalizing symptoms (10). Given the high risk that rumination will lead to physical and mental problems, particularly depression and self-injury in adolescents (11), it is of importance to identify and prevent children’s rumination in their early adolescence during the post-COVID-19 epidemic era.

Maladaptive parenting practices can account for individuals’ rumination. Studies have found that harsh parenting (12, 13), inter-parental conflict (14), parental divorce (15), poor mother-adolescent relationship (5), parental over-protection (9, 16), and parental demandingness (17) in childhood are positively related to rumination in early adulthood. Conversely, adaptive parenting practices can protect children from experiencing rumination and depression. Evidence from studies in recent years suggests that positive parenting (11), parental positive communication (18), parental acceptance/warmth (19), etc. can protect children from rumination. Although valuable, these works have mainly explored the links between parenting and rumination as well as their process mechanisms by focusing on the joint effects of parenting or on the separate effects of parenting by fathers or/and mothers (12). Researchers should be aware that the parenting practices of fathers and mothers are not isolated from each other, but instead interact with each other. It is necessary to consider the issue of the interaction between fathers and mothers in co-parenting conditions in the family system and their complex influence on their offspring. According to family systems theory (20), children’s socialization and internalizing and externalizing problems are susceptible to the interdependence of family members, especially the interactions between parents (21). Fortunately, the parental acceptance-rejection theory[PARTheory (22, 23);] provides a valuable analytical framework for dealing with this issue, and researchers have achieved fruitful outcomes from this approach (24, 25). However, most of these studies have mainly focused on the relationships between parental rejection or/and parental warmth and children’s depression from a holistic perspective (26, 27), or the effects of paternal or maternal rejection/warmth on children’s depression, independently (28), without exploring the interaction effect between paternal and maternal parenting on adolescents’ rumination. A notable exception is Miranda et al. (29), who explored the relationships between parental acceptance-rejection and adolescent maladjustment by combining the fathering and mothering together. Although they focused on the differences between parenting consistency and inconsistency as well as their effect on adolescent maladjustment, their study still inspired us to consider the question of the complex relationship pattern between parenting discrepancy and adolescent rumination from the perspective of co-parenting and family systems. Thus, to further clarify the issue, we decided that a more systematic perspective and study design should be conducted to address this gap.

## 2 Construction of the model

### 2.1 Parental coparenting patterns with inconsistency between mothering and fathering in Chinese families

In families, fathers and mothers often prefer to approach parenting practice in different ways (30) [see also (31)for a review], and this phenomenon shows a more complex style in China. For example, in traditional Chinese culture, fathers showed less concern for their children and used harsher discipline than mothers did (32). By contrast, mothers were likely to show more concern and warmth to children (33, 34). This parenting style is called “strict father, kind mother” (*yan fu, ci mu*) (21). However, more recently, there has been a shifting trend in urban Chinese families toward a “strict mother, kind father” (*yan mu, ci fu*) constellation (21), which means that mothers are more likely to show strict behavioral control and issue direct commands and physical punishment than fathers are (35). In summary, there are two patterns of parenting inconsistency constellation (i.e., “strict father, kind mother” and “strict mother, kind father”) in contemporary Chinese families.

### 2.2 The interacting effect of parental and maternal rejection-warmth in predicting adolescents’ rumination

The PARTheory asserts that parental acceptance/warmth and parental rejection are two termini of a continuum in which expressions and perceptions range from aversion, neglect, lack of affection, hostility, aggression, indifference, and coldness by parents to warmth, affection, care, comfort, concern, nurturance, support, or simply love that children can experience from their parents (36–38). Parental rejection leads to psychological and behavioral maladjustment in adolescents and adults (37, 39). “Children who perceived being rejected by their parents are more likely to a) have a weak sense of self-esteem and self-adequacy, b) be emotionally unresponsive or unstable; c) have a pervasive negative worldview” (37); and d) psychological disorders (36). PARTheory further posits that all humans have a psycho-biological need to feel supported and attached to their care-givers (especially the parents), and such secure attachment leads to greater trust and emotional security about the world and themselves (25), which is called “internal working model”(IWM) by Bowlby (40, 41). IWM is a schema about an individual herself/himself and others that would guide the individual’s daily functioning, particularly in the interpersonal contexts (41). Quirk et al. concluded that the mechanisms underlying the associations between parental rejection and rumination is the impairment in cognitive and emotional functioning in children caused by rejection from their parents (26). According to PARTheory, the functioning impairment arises because children’s need for support is denied and they internalized an insecure IWM in themselves, characterizing alienation from their harsh parents and excessive self-anxiety (23). One research on the antecedents of ruminative brooding and worry has supported this view (17).

Not all individuals who experience parental rejection in childhood engage in rumination. This indicates that it is necessary to identify moderators of the association between perceived parental rejection and rumination. Based on PARTheory, parental acceptance/warmth is expected to be one of the most prominent factors. Unlike parental rejection, parental acceptance is associated with multiple positive psychological outcomes, such as a strong sense of self-esteem, good emotional stability, a positive worldview, and the like (23), and researches had confirmed these arguments (19, 42). Also, further research has shown that paternal warmth can help to reduce or even prevent depression and rumination in adolescents (27, 28). According to Rohner’s (23, 43) claim, parental warmth meet children’s psychological and biological need to feel supported and attached to their care-givers, and children develop a positive IWM in the secure attachment relationship. Thus, children will be more likely to develop a positive world view toward themselves and others, and will be less likely to engage in rumination.

Parental warmth would buffer the damaging effects of parental rejection on children. In literature, researchers found that parental rejection leads to a number of internal and external problems in adolescents (44), while parental warmth, as a protector, helps to protect against future anxiety and depressive symptoms in adolescents (28). According to a postulation of PARTheory, the likelihood of children being able to cope with perceived rejection from one parent is enhanced by the presence of a supportive, warm, alternate caregiver or attachment figure (23). According to family resilience theory (45), resilience arises in terms of an interplay of risk and protective processes, involving individuals, families, and larger sociocultural influences (45), and children suffering from a psychological crisis due to harsh treatment from one parent may gain resilience from the warmth of the other parent in the co-parenting system. A cross-cultural longitudinal study covering eight countries by Lansford et al. explored the interaction between parental corporal punishment and maternal warmth in predicting adjustment problems in children (46). Its results show that children with a high level of maternal warmth who suffered corporal punishment were associated with a lower level of adjustment problems. Another study by Fahmy confirmed more directly that warmth from one parent does buffer against rejection from the other for college students (47). Specifically, children who perceived rejection from one parent did show a significantly lower level of internalized shame under the condition of a high level of acceptance from the other parent than did the children under the condition of a low level of acceptance (47). Therefore, it is reasonable to expect that warmth from one parent may moderate the effect of the rejection of the other parent in causing adolescent rumination.

### 2.3 The conditional effect of adolescents’ gender in the moderating models

The gender differences in adolescents’ rumination have been discussed fruitfully. Studies to date have shown that girls exhibit a higher level of ruminative state compared to boys (48). Especially in adolescence, girls confront a quick and intense change in their mental and physical state; they are more sensitive to these changes when they have a vulnerable self-concept and a strong sense of apprehension (49). As for cognitive style, girls possess a more negative cognitive disposition than boys, and thus engage in more severe rumination (6). For example, compared with boys, girls in early adolescence evaluate stressors to be more negative (50), and more easily engage in sadness rumination when confronted with the same external pressures as boys (10, 51), such as perceiving parental rejection or other harsh parenting practice (52). Thus, theoretically, when perceiving the same parenting practices, female adolescents and male adolescents would be expected to experience different psychological consequences.

The effect of the interaction between parental rejection and parental warmth in predicting adolescents’ rumination should be differentiated by gender. For boys, perceived maternal warmth (MW) would be expected to moderate the link between perceived paternal rejection (PR) and adolescents’ rumination, while this would not be expected for the girls. Not only are boys more resistant to the effects of harsh parenting, but also they gain more resilience from parents’ love and warmth than girls do. One study has shown that for girls, perceived maternal rejection gives rise to more internalized symptoms (i.e., sense of shame) than do boys (47). Consequently, when taking the differences between mothering and fathering into account, as well as children’s gender, it is reasonable to expect that the interaction between rejection from one parent and warmth from another parent will vary by children’s gender.

The children’s gender difference has always been a traditional issue in Chinese parenting practice, including into the present day. In traditional China, the *son preference* was a prominent social phenomenon by which parents valued and favored sons over daughters (53), and the same condition holds today (54). The son preference is reflected not only in prenatal gender selection and providing more nursing time and medical resources for boys but also in differences in parenting practice toward children of different genders (55). Parents who hold the concept of a son preference will direct more soft words and emotional attention to the sons, which leads to a good psychological experience (i.e., perceived parental warmth) for boys but a bad one (i.e., perceived parental rejection) for girls (1, 32). With regard to the female children’s innate sensitivity and vulnerability in their response to harsh parenting practices, we supposed that daughters who experienced parental rejection would be more likely to suffer rumination than sons.

### 2.4 The current study

**Figure 1:**
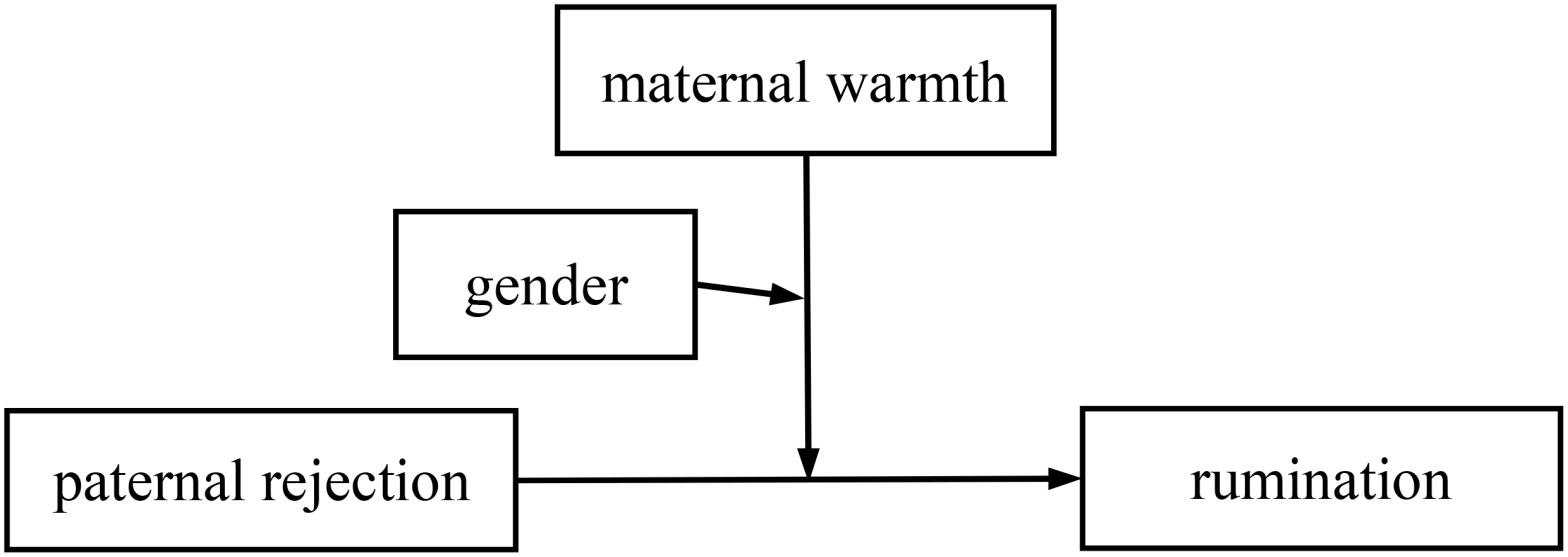
A conditional moderating model of maternal warmth and gender in the link between paternal rejection and adolescents’ rumination.

**Figure 2:**
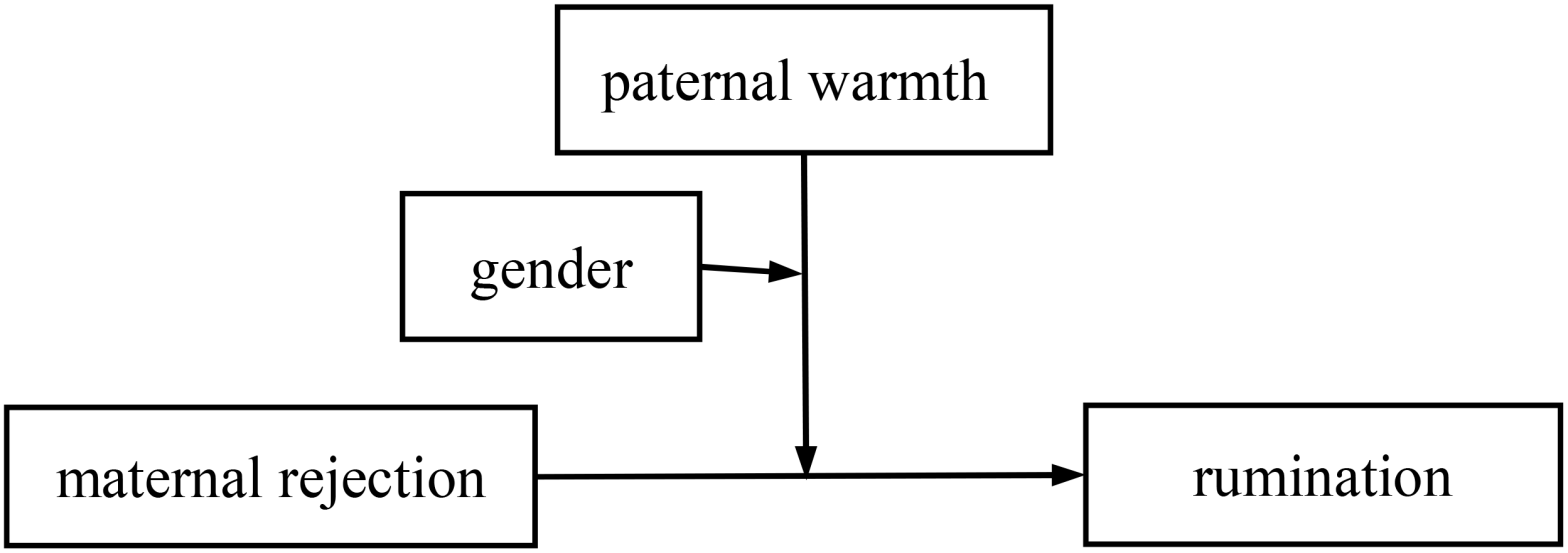
A conditional moderating model of paternal warmth and gender in the link between maternal rejection and adolescents’ rumination.

This study included perceived parental rejection and parental warmth as two family-related environmental psychological factors in constructing two conditional moderation models with children’s gender as a moderator (see **Figures 1**, **2**) to explore the underlying mechanisms by which co-parenting practices could predict rumination in Chinese early adolescents during the post-COVID-19 era. Specifically, we put forward the following hypotheses: 1) perceived PR and maternal rejection (MR) are positively associated with adolescents’ rumination, 2) perceived paternal warmth (PW) and MW are negatively associated with adolescents’ rumination, 3) perceived MW moderates the relationship between perceived PR and adolescents’ rumination, 4) perceived PW moderates the relationship between perceived MR and adolescents’ rumination, and 5) the interaction of perceived parental rejection and parental warmth in predicting adolescents’ rumination is moderated by the adolescents’ gender.

## 3 Methods

### 3.1 Participants

Participants were recruited from two middle schools in Chongqing and Changsha, China by convenience cluster sampling. A total of 1171 students participated in this survey. We eliminated subjects who did not come from a two-parent family. After cleansing the raw data (participants who did not entirely and correctly complete all the measures and scored out of the range of [-3, 3]), we obtained a sample of 910 adolescents. The mean age of the students was 13.63 (*SD* = 1.06 years; range = 11–16 years). Among this sample, 48.50% were female and 50.50% were male, with nine missing values. 349 (38.4%) were *only-child* and 542 (59.6%) were not *only-child*, with nineteen missing values. As for the *Domicile*, 584 (64.2%) were *urban* residents, while 281 (34.9%) were *rural* residents, with forty-five missing values. For the *Grade* information, 338 (37.1%) were 7^th^ graders, 259 (28.5%) were 8^th^ graders, and 312 (34.3%) were 9^th^ graders, with one missing value.

### 3.2 Measures

#### 3.2.1 Perceived parental rejection and warmth

Perceived parental rejection and perceived parental warmth were measured by the paternal rejection sub-scale and maternal warmth sub-scale from the Short form Egna Minnen av Barndoms Uppfostran for Chinese scale [S-EMBU-C (56);], which was created as a revision of the EMBU (44, 57). Compared to the parental acceptance-rejection Questionnaire, the current scale is more suitable for adolescents to report their perceived parenting style (44). All items were rated on a 4-point Likert scale ranging from 1 (never) to 4 (always). Scores were calculated by summing up all the response scores, with higher scores indicating higher levels of parental rejection/warmth. This measure has demonstrated good reliability and validity with Chinese students (56).

The parental rejection sub-scale with 6 items mainly evaluates the degree to which the child perceives a lack of love, affection, and comfort from their fathers or mothers (56). Children rate their fathers and mothers separately on the items, such as *“*my parents would punish me hard, even for trifles (small offenses)”, and “my parents treated me in such a way that I felt ashamed.” In the current study, the Cronbach’s α for the paternal and maternal rejection sub-scales were 0.790 and 0.789, respectively. The parental warmth sub-scale with 7 items measures the emotional warmth a child perceives coming from their mother or father. Sample items are, “my parents praised me”, and “my parents tried to spur me to become the best”. Each child separately rated his or her father and mother. In the current study, Cronbach’s α for the paternal warmth and maternal warmth sub-scales were 0.865 and 0.861, respectively.

#### 3.2.2 Rumination

Rumination was measured through the Chinese version of the Ruminative Responses Scale (58), which was adapted from the original Ruminative Responses Scale (59). This scale consists of 22 items and contains three factors, namely brooding (e.g., thinking “Why do I always react this way?”), symptom rumination (e.g., “thinking about how alone I feel.”), and reflection (e.g., “going somewhere alone to consider how I feel.”). Participants give answers on a 4-point Likert scale ranging from 1(never) to 4(always). A higher total score indicates a higher level of rumination. The original version exhibits good reliability[Cronbach’s α is 0.89 in (59)]. Cronbach’s α for the Chinese version of the scale was 0.87 in the current study.

### 3.3 Procedures

The study protocol was approved by the first author’s University Scientific Research Department and has been executed in conformity with ethical standards laid down in the 1964 Declaration of Helsinki and its later amendments. This research team completed collecting data from April through June, 2021. During data collection, a researcher or an assistant read instructions to the whole class ahead of the survey. All participants were informed that their participation was voluntary, and oral informed consent was obtained from students and their parents. Later, the students filled out questionnaires regarding demographics, parental rejection, parental warmth, and rumination in the classroom.

### 3.4 Data analysis

Before the formal analysis, we deleted extreme values beyond the range of ±3 standard deviations and filled in missing values of the measured variables using the linear interpolation method. Descriptive statistics and correlations analysis were then conducted using SPSS 26.0 micro software. To minimize multi-collinearity, we centered all the predictors and observed variable (60). After that, we examined the multi-collinearity of the two conditional moderation models separately; the results showed that all tolerances and variance inflation factors (VIF) were within normal limits (tolerance = [0.854, 0.993]; VIF = [1.007, 1.171]), indicating no severe issue of multi-collinearity among the predictors (61). We then used Hayes’s (2013) PROCESS 3.5 macro (Model 3) to investigate whether parental warmth and gender moderated the link between parental rejection and adolescents’ rumination in the two conditional moderating models (62). Bias-corrected bootstrapping based on 5000 samples was used to estimate the standard errors of conditional effects. Meanwhile, we calculated conditional direct effects to test whether the direct effect of rejection from one parent on rumination varied at different levels of the moderating variables (i.e., maternal warmth, paternal warmth, and gender). Simple slope graphs were drawn to illustrate this effect in an intuitive fashion. Given that adolescents’ age, status of only-child, and grade might correlate to rumination (48), we controlled these demographic variables in our statistical analyses. Note that, for convenience of regression analysis, all the categorical variables were recoded into dummy variables with values of [0,1].

## 4 Results

### 4.1 Descriptive statistics

**Table 1** presents the means, standard deviations, and correlations for all variables. Adolescents who perceived more PR or more MR (*p* < 0.001) were more likely to ruminate (*r_s_* = 0.326, and 0.343 respectively, *p_s_* < 0.001). Perceived PW and MW were both negatively related to rumination (*r*_s_ = -0.184, and -0.125 respectively, *p*_s_ < 0.001). Meanwhile, the results also indicate that adolescents’ gender and 8^th^ grade were positively related to rumination (*p*_s_ < 0.01). More details can be found in **Table 1**.

**Table 1:** Means, standard deviations, and correlations for study variables.

	1	2	3	4	5	6	7	8	9	10	11
1.Age	—										
2.Gender ^a^	-0.005	—									
3.Only-child ^b^	-0.059	0.072^*^	—								
4.Domicile ^c^	0.163^***^	0.049	0.116^***^	—							
5.8^th^ grade ^d^	0.096^**^	0.028	0.025	0.132^***^	—						
6.9^th^ grade ^e^	0.708^***^	0.015	-0.088^**^	0.058	-0.454^***^	—					
7.Paternal rejection	0.074^*^	-0.025	0.099^**^	0.101^**^	0.172^***^	-0.015	—				
8.Maternal rejection	0.071^*^	0.020	0.095^**^	0.075^*^	0.148^***^	-0.011	0.719^***^	—			
9.Paternal Warmth	-0.095^**^	-0.088^**^	-0.143^***^	-0.135^***^	-0.151^***^	0.019	-0.417^***^	-0.374^***^	—		
10.Maternal Warmth	-0.107^**^	-0.065^*^	-0.136^***^	-0.136^***^	-0.166^***^	-0.001	-0.321^***^	-0.462^***^	0.825^***^	—	
11.Rumination	0.016	0.098^**^	0.056	-0.018	0.134^***^	-0.037	0.326^***^	0.343^***^	-0.184^***^	-0.125^***^	—
*M*	13.63	0.490	0.608	0.325	0.284	0.343	9.625	10.011	18.710	19.668	43.150
*SD*	1.057	0.498	0.484	0.457	0.451	0.475	3.555	3.615	5.208	5.030	10.407

n = 910. ^a^ gender was recoded as male = 0 and female = 1. ^b^ only-child status was recoded as an only-child = 0 and not an only-child =1. ^c^ domicile was recoded as town =1 and village = 1. grade was recoded as two dummy variables, with ^d^ 8^th^ grade recoded as 8^th^ grade =1 and else =0, and ^e^ 9^th^ grade recoded as 9^th^ grade =1 and else = 0. ^*^ *p* <.05. ^**^ *p* <.01. ^***^ *p* <.001.

### 4.2 The regression paths and moderating effects in the conditional interaction models

We first tested the conditional moderation effect model with perceived MW and adolescents’ gender moderating the link between perceived PR and adolescents’ rumination (see **Figure 1**). The results of the regression equations test, shown in **Table 2**, indicate that all the predictors significantly predicted adolescents’ rumination (*F*(12, 897) = 11.929, *p* <0.001, *R* ^2^ = 0.138), and they accounted for 13.8% of the variance in rumination. Specifically, in this regression model, PR (*b =* 0.814, *SE* = 0.134, *t* = 6.078, *p* < 0.001) and the PR × MW interaction both significantly predicted rumination (*b = -*0.066, *SE* = 0.024, *t* = -2.734, *p* = 0.006), and both participants’ gender and 8^th^ grade were significantly related to rumination (all *p*_s_ *<*0.05). However, age, only-child status, and the PR × MW × gender interaction were not significantly related to rumination (all *p*_s_ >0.05). Furthermore, according to the results of the bootstrapping test, whether adolescents who experienced PR would tend to ruminate or not depended on whether they had a low or high level of perceived MW (Boot effect = -0.006, Boot SE = 0.029, 95% CI = [-0.124, -0.010]), and this interaction pattern (PR × MW interaction) was independent of adolescents’ gender (PR × MW × gender interaction; Boot effect = -0.063, Boot SE = 0.040, 95% CI = [-0.015, 0.140]).

**Table 2:** All regression paths and bootstrapping test of the conditional moderating model with paternal rejection, maternal warmth, gender, and rumination in adolescents.

Predictors	Regression paths (Criterion: Rumination)				Bootstrapping Test			
	*b*	*SE*	*t*	*p*	Effect	BootSE	LLCI	ULCI
Age	-0.541	0.583	-0.927	0.354	-0.541	0.581	-1.678	0.583
Gender	2.544	0.685	3.715^***^	<0.001	2.544	0.697	1.149	3.926
Only-child	0.490	0.685	0.715	0.475	0.490	0.681	-0.857	1.822
Domicile	-1.693	0.733	-2.311^*^	0.021	-1.693	0.719	-3.117	-0.309
8^th^ grade	2.992	1.102	2.716^**^	0.007	2.992	1.060	0.905	5.090
9^th^ grade	1.501	1.452	1.034	0.302	1.501	1.409	-1.26	4.252
PR	0.814	0.134	6.078^***^	<0.001	0.814	0.147	0.516	1.092
MW	-0.020	0.097	-0.209	0.834	-0.020	0.108	-0.235	0.194
PR×MW	-0.066	0.024	-2.734^**^	0.006	-0.066	0.029	-0.124	-0.010
PR×Gender	0.168	0.197	0.856	0.392	0.168	0.211	-0.230	0.597
MW×Gender	0.012	0.137	0.085	0.932	0.012	0.146	-0.276	0.295
PR×MW× Gender	0.063	0.035	1.814	0.070	0.063	0.040	-0.015	0.140
*R^2^*	0.138							
*F*(12, 897)	11.929^***^							

n = 910. Variables of Age, Only-child, Domicile, and Grade were controlled.

PR, paternal rejection; MW, maternal warmth; LLCI, lower limit of confidence interval; ULCI, Upper limit of confidence interval. ^*^ *p* <.05. ^**^ *p* <.01. ^***^ *p* <.001.

We then examined the conditional moderation model with perceived PW and gender moderating the link between perceived MR and adolescents’ rumination (see **Figure 2**). The results of the regression test show that all the predictors significantly predicted adolescents’ rumination [*F*(12, 897) = 13.248, *p* <0.001, *R* ^2^ = 0.151], accounting for 15.1% of the variance in rumination. Specifically, adolescents with more MR were more likely to ruminate (*b =* 0.758, *SE* = 0.139, *t* = 5.444, *p* < 0.001), and whether children who experienced MR would tend to ruminate or not depended on whether they perceived a low or high level of perceived PW (MR × PW interaction) (*b =* -0.052, *SE* = 0.023, *t* = -2.278, *p* = 0.023). The association between the MR × PW interaction and adolescents’ rumination significantly varied by adolescents’ gender (*b* = 0.103, *SE* = 0.034, *t* = 2.980, *p* = 0.003). Also, adolescents’ gender, domicile, and 8^th^ grade all significantly predicted rumination (all *p_s_* <0.05). Furthermore, the results of the bootstrapping test indicated that whether adolescents who experienced PW would tend to ruminate or not depended on a low or high level of perceived PW (PR × MW interaction; Boot effect = -0.052, Boot SE = 0.026, 95% CI = [-0.103, -0.001]), and the predictive association between PR × MW interaction and children’s rumination varied with children’s gender (Boot effect = 0.103, Boot SE = 0.037, 95% CI = [0.029, 0.177]). See **Table 3** for more details.

**Table 3:** All regression paths and bootstrapping test of the conditional moderating model with maternal rejection, paternal warmth, gender, and rumination in adolescents.

Predictors	Regression paths (Criterion: Rumination)				Bootstrapping Test			
	*b*	*SE*	*t*	*p*	Effect	Boot*SE*	LLCI	ULCI
Age	-0.663	0.579	-1.144	0.253	-0.663	0.574	-1.802	0.462
Gender	2.488	0.693	3.590^***^	<0.001	2.488	0.699	1.123	3.831
Only-child	0.316	0.680	0.464	0.643	0.316	0.702	-1.087	1.679
Domicile	-1.581	0.726	-2.178^*^	0.030	-1.581	0.722	-2.993	-0.167
8^th^ Grade	3.089	1.087	2.843^**^	0.005	3.089	1.044	1.082	5.153
9^th^ Grade	1.653	1.442	1.146	0.252	1.653	1.415	-1.123	4.435
MR	0.758	0.139	5.444^***^	<0.001	0.758	0.156	0.451	1.061
PW	-0.183	0.096	-1.919	0.055	-0.183	0.108	-0.394	0.037
MR×PW	-0.052	0.023	-2.278^*^	0.023	-0.052	0.026	-0.103	-0.001
MR×Gender	0.355	0.199	1.530	0.126	0.304	0.212	-0.107	0.723
PW×Gender	0.146	0.135	1.083	0.279	0.146	0.146	-0.138	0.438
MR×PW× Gender	0.103	0.034	2.98^**^	0.003	0.103	0.037	0.029	0.177
*R^2^*	0.151							
*F*(12, 897)	13.248^***^							

n = 910. Variables of Age, Only-child, Domicile, and Grade were controlled.

MR, maternal rejection. PW, paternal warmth; LLCI, lower limit of confidence interval; ULCI, Upper limit of confidence interval. ^*^ *p* <.05. ^**^ *p* <.01. ^***^ *p* <.001.

**Figure 3:**
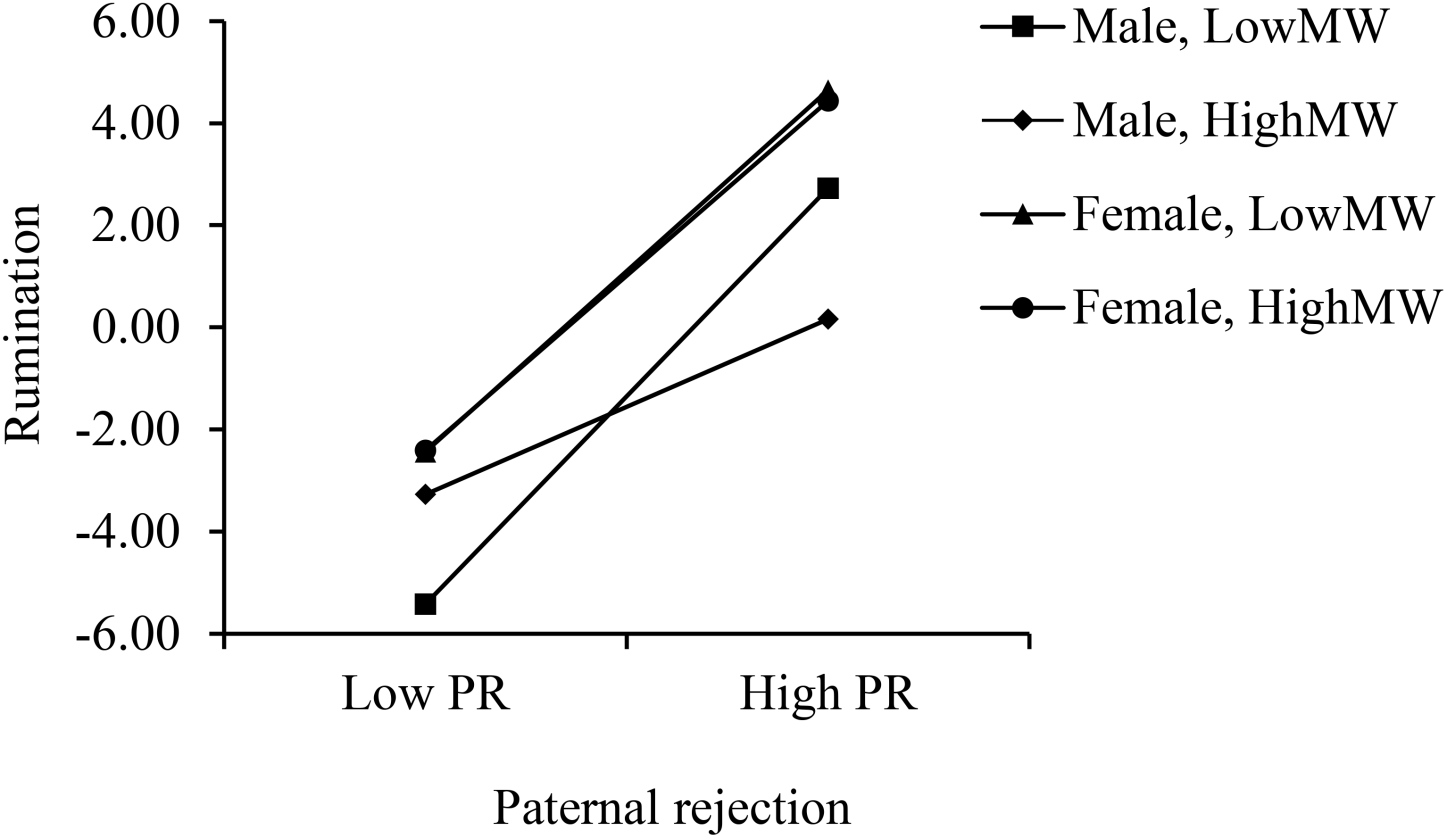
The conditional moderating effect of maternal warmth and paternal rejection in predicting adolescents’ rumination by gender. PR, paternal rejection; MW, maternal warmth.

Simple slope tests were then conducted to demonstrate the interaction patterns of PR × MW for different adolescents’ genders. As shown in **Figure 3**, for the boys, the PR × MW interaction significantly predicted rumination (*b*_simple_ = -0.066, *F* = 7.474, *p* = 0.006). However, this predictive relation was not statistically significant for girls (*b*_simple_ = -0.004, *F* = 0.020, *p* = 0.887). Moreover, the results of the conditional effects test revealed that the conditional effect of perceived PR was significant under all conditions of ±1 SD MW for both gender (all *p*_s_ < 0.05). Specifically, the effect of perceived PR under the condition of a low level of MW (1 SD below the mean score) was the largest for boys (effect = 1.145, *SE* = 0.148, *t* = 6.829, *p* < 0.001), and was 0.998 for girls (*SE* = 0.169, *t* = 5.888, *p* < 0.001); the effect was 0.962 under the condition of +1SD MW for girls (*SE* = 0.208, *t* = 4.633, *p* < 0.001), and it was lowest of all under the condition of +1SD MW for boys (effect = 0.483, *SE* = 0.193, *t* = 2.509, *p* = 0.012).

**Figure 4:**
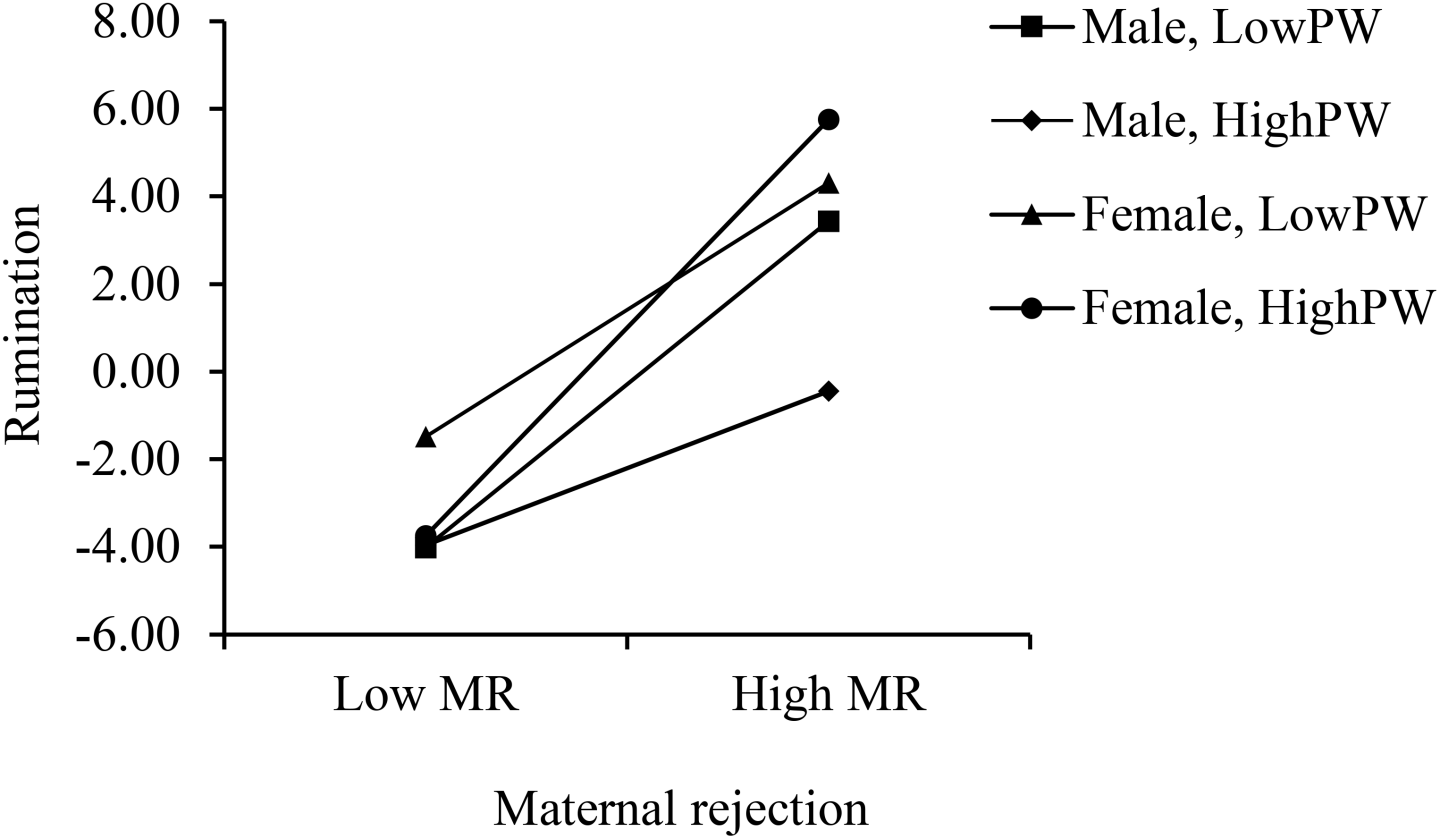
The conditional interaction between paternal warmth and maternal rejection in predicting adolescents’ rumination by gender. MR, maternal rejection; PW, paternal warmth.

**Figure 4** depicts the results of simple slope tests for the patterns of the MR × PW interaction with different adolescents’ genders. For male adolescents, the relation between MR × PW interaction and rumination was significant (*b*_simple_ = -0.052, *F* = 5.187, *p* = 0.023). However, this relation was not statistically significant for female adolescents (*b*_simple_ = 0.049, *F*= 3.775, *p* = 0.052). The results of the conditional effects test revealed that the conditional effect of MR was significant under all conditions of ±1 SD PW for both boys and girls (all *p*_s_ < 0.05). Specifically, the effect of perceived MR under the condition of -1 SD PW was 1.029 for boys (*SE* = 0.167, *t* = 6.149, *p* < 0.001), and 0.802 for girls (*SE* = 0.159, *t* = 5.058, *p* < 0.001). The effect of perceived MR was largest under the condition of +1SD PW for girls (effect =1.315, *SE* = 0.223, *t* = 5.909, *p* < 0.001), and it was lowest of all under the condition of +1SD PW for boys (effect = 0.486, *SE* = 0.198, *t* = 2.456, *p* = 0.014).

## 5 Discussion

The current study proposed two conditional moderation models to explore how perceived rejection of one parent interacted with perceived warmth from the other parent in predicting adolescents’ rumination and whether this interaction varied by children’s gender under the dual framework of PARTheory and family resilience theory. In general, the study hypotheses were at least partially confirmed. As expected, adolescents who perceived more PR and/or MR were more likely to engage in rumination, while adolescents who perceived more PW and/or MW were less susceptible to rumination. Meanwhile, for the combination of PR and MW, if boys who suffered PR perceived more MW, they were less likely to ruminate, while girls who perceived more PR were more susceptible to rumination regardless of their perception of MW. For the combination of MR and PW, if boys who suffered MR perceived more PW, they were less likely to ruminate, while girls who perceived more MR were more susceptible to rumination regardless of their perception of PW than their male counterparts.

### 5.1 Main findings

There are three main findings of this study. First, several correlations in the current study were consistent with the hypotheses. Specifically, the results indicated that perceived PR and perceived MR were positively related to rumination in adolescents, while perceived PW and perceived MW were negatively correlated with it. These results are consistent with those of a previous study (63), confirming the destructive role of perceived parental rejection and the constructive role of perceived parental warmth in children’s mental health, and it is also consistent with the basic tenet of PARTheory (38).

Second, we found that perceived MW moderated the link between perceived PR and adolescents’ rumination. Adolescents who perceived rejection from their fathers but warmth from their mothers were not inclined to ruminate. Conversely, adolescents who experienced less warmth from their mothers were more likely to be ruminative. As explained by the PARTheory, maternal acceptance and warmth bring comfort and inner security to the children and alleviate the perceptions of coldness and anxiety caused by the father’s rejection (38). Furthermore, this protective role of mothers in the links between children and harmful paternal roles maintains ideal conformity with the family resilience theory (45), which implies that mothers’ caring and love stimulate children’s potential to recover from harsh fathering practices. Also, former studies have greatly validated the mothers’ preventive role in protecting children against variety of negative circumstantial factors (64, 65). Notably, the favorable effect of perceived MW only applied to the male adolescents, excluding the females. This indicates that the female adolescents were more vulnerable to their parents’ coldness and neglect than the boys, that is, they were more difficult to recover from the hurt of the father’s rejection, regardless of the mother’s warmth. Despite limited direct evidence, former studies have confirmed the disadvantages of girls in dealing with harsh parenting practice (66), and their innate vulnerability to rumination (5). Thus, the current study initially identified a critical condition for the interaction system of strict father-kind mother-child within the framework of family resilience theory.

Third, the results also indicated a significant interaction between perceived MR and perceived PW in predicting children’s rumination in their early adolescence. That is to say, the perception of PW altered the extent to which perceived MR was related to adolescents’ rumination, and this interaction effect varied by the adolescents’ gender. For boys, adolescents with high PW perception showed a weaker association with rumination. This finding is in accordance with the tenets of PARTheory and family resilience theory, which posit that perceived warmth and acceptance from fathers protect boys who have suffered perceived MR from being ruminative by strengthening their resiliency (67). Studies have also confirmed the positive effect of fathers’ warmth, not just mothers’ warmth, on children from their early childhood (68, 69), which likewise supports the claims of PARTheory. However, the PW×MR interaction showed an unusual pattern for girls. Specifically, girls who experienced more PW showed a stronger association between perceived MR and rumination compared with those who experienced less PW. This implies that perceived fathers’ warmth causes girls to be more susceptible to rumination when they perceive their mothers as cold. From the perspective of attachment theory, mothers play a more critical role than fathers in the secure parent-children attachment relationship. This is mainly because mothers tend to be more caring, loving, and magnanimous, and thus deliver more warmth and security to children. Especially in Chinese families, mothers mainly act as caregivers and tend to provide their children with affection, warmth, verbal induction, daily care, and assistance with school assignments (32), and the maternal parenting model becomes the child’s psychological schema for the maternal role. With this schema, children expect and seek warmth from their mothers, but if they do not attain it, they feel a psychological gap and develop a sense of maltreatment. Even worse, for girls, the sense of hurt from their mother cannot be soothed by a warm father. Quite the opposite, fathers’ warmth intensifies children’s resentment toward their mothers and dislike of their fathers. This inverted combination of parenting practice contradicts the Chinese *yan fu, ci mu* (strict father, kind mother) constellation (21), and it hurts female adolescents more than male adolescents. A former study used latent profile analysis approach to identify four patterns of co-parenting by Chinese parents, and the results showed that children with a “kind father, strict mother” constellation suffered more severe externalized and internalized problems than those with a constellation of “kind mother, strict father” (21). It seems that Chinese children are more comfortable with the co-parenting constellation of “strict father, kind mother” than with the “strict mother, kind father” constellation, and one study has shown that girls are indeed more susceptible to harsh mothering than to harsh fathering (70). This may account for the extraordinary result of the current study.

### 5.2 Research strengths and implication

The current study makes several theoretical contributions to the literature. First, it advances the literature on co-parenting with two diametrically opposite parenting practices between fathers and mothers by exploring their interaction effect on adolescents’ rumination. Within the framework of family systems theory, the findings of the current study not only comfortably support the PARTheory and family resilience theory, but also depict a relatively clear picture of parental interaction as well as its association with children’s mental health, separately for female and male adolescents in Chinese families. As a general rule, perceived parental warmth serves a critical role in protecting children from being ruminative, while perceived parental rejection does the opposite. When putting the static categorization of parental rejection-warmth into the framework of a family resilience system, we found that perceived parental warmth also indeed alleviated the detrimental influence of parental rejection on children. Thus, this study optimizes the PARTheory by introducing a theoretical dimension of dynamic interaction processes into the current static model. With the aid of this perspective, people will hopefully see more complex patterns of dynamics regarding parental warmth and rejection between mothers and fathers, rather than classifying them statically (21). Furthermore, we confirmed the gender differences in adolescents’ rumination under different parenting constellations (i.e., strict father with kind mother *vs.* strict mother with kind father) in contemporary Chinese families. Male adolescents who experienced MR had greater resilience when they perceived high PW, while this did not apply to females. Moreover, girls who experienced MR had surprisingly worse mental outcomes in the high PW condition than under low PW. The same was not observed for boys, who were still protected by PW. All these findings add new experience materials and theoretical clues for researchers in exploring the relationship between co-parenting and adolescents’ mental health in Chinese family culture.

The current study also has clear practical implications. Parents should be more loving, caring, etc. toward their children, thereby delivering a sense of warmth to their children, especially before their early adolescence. In light of our findings, a pair of parents can also conduct different parenting practices toward their sons or daughters. For example, given that damage for girls by parental rejection could be profound and irreversible, both the mother and the father should provide their daughter with more care and warmth, and less indifference and neglect. In addition, one parent should show their son more acceptance and warmth, if the son has a sense of rejection from the other parent. Mental health counselors will gain more specific and targeted documents about the family information of adolescents with depression. Middle school teachers can be more aware of how to communicate with parents to improve their parenting practices with ruminative adolescents.

How can be parents accompanied to be less rejecting and more warm? Maybe some validated programs could help. Particularly, based on self-determination theory (71), satisfying parental needs would lead them to be less rejecting/controlling. For instance, several studies have demonstrated that parents’ daily experiences of need satisfaction were related uniquely to parental autonomy-supportive parenting (72), psychological need satisfaction was negatively related to parental conditional regard (73), and parental autonomous motivation for involving in children’s homework was positively related to parents’ positive emotion as well as their children’ positive self-efficacy for homework (74). Thus, psychological and educational interventions based on enhancing parental needs fulfillment and autonomy motivation may enhance parental warmth and reduce parental rejection. School teachers and family counselors can also use these approaches to address parent-child relationship issues and improve children’s mental health indirectly.

### 5.3 Limitations and perspectives

The current study has several limitations that point to directions for future research. First, the cross-sectional design made it impossible to draw a causal deduction concerning the relations among the study variables. For example, children who ruminate may tend to perceive rejection from their parents (75). Thus, future studies should apply a longitudinal or experimental design to explicate the causal links among the main variables in the future. Second, the retrospective self-report measurement of parenting style by children may be inaccurate because a long time has passed. In addition, the retrospective memory bias and social desirability effect may bias self-reports of perceived parental warmth-rejection and rumination (76). Thus, objective measurements in future studies, such as parent-rating measurements or an experimental method, would be helpful; the parental self-reported parental acceptance-rejection questionnaire would fit better with the PARTheory than the EMBU scale used in current study (36). Third, we did not include other variables such as family socioeconomic status, parental stress as well as children’s personality characteristics, which also contribute to adolescents’ rumination (16, 77) and could be controlled in future research on analogous topics. Also, time spent with parents would also affect the children’s perception of parental warmth-rejection (78) as well as their symptoms related to rumination (79), and there may be complex interactions between them. Last but not least, the current study has just focused on two coparenting inconsistency constellations neglecting other coparenting consistency constellations (i.e., strict father and strict mother and also kind father and kind mother constellations). Prior research found that there are various patterns of coparenting constellations, such as both parental warmth and both parental rejection (29), using a person-centered approach(e.g., cluster analysis method and latent profile analysis method), and we will also apply a person-centered approach to further explore the patterns of coparenting constellations with parental warmth and rejection in Chinese families with junior high school adolescents, as well as to examine their relationships with adolescent rumination. Given that the current findings were derived from a Chinese sample, further examination is needed to determine whether the results can be applied to other cultural populations, especially for the moderating effect of gender in the relations between perceived parental warmth-rejection and rumination of adolescents from diverse cultural backgrounds.

## 6 Conclusions

Perceived Parental rejection and parental warmth co-exist in the Chinese family system, and they exert an interactive effect on adolescents’ rumination depending on their gender. For boys, perceived warmth by one parent will protect them from engaging in rumination by attenuating the damage from rejection perception by the other parent. However, perceived parental warmth will be invalidated in preventing rumination for female adolescents once they perceive to be rejected by another parent. It implies that both parents should be more accepting, caring, considerate, and warm toward their daughters, and it is also in line with the tradition and status quo of parenting in Chinese families. These findings have implications for parental co-parenting practices in Chinese families with early adolescents and are useful for middle school teachers as well as adolescents psychological counselors, and validated programs of psychological and educational interventions based on enhancing parental needs fulfillment and autonomy motivation should be established to upgrade parental warmth and reduce parental rejection.

## Data availability statement

The original contributions presented in the study are included in the article/**Supplementary Material**. Further inquiries can be directed to the corresponding authors.

## Ethics statement

The studies involving humans were approved by Research Department of Changsha Normal University. The studies were conducted in accordance with the local legislation and institutional requirements. Given the low-risk nature of this survey for adolescents, written parental consent was not collected. However, prior to the survey, the researcher informed the parents of the students about the nature of the study, its purpose, the terms of the confidentiality agreement, and other content terms through the WeChat group of the students’ parents, and verbal informed consent was obtained from all parents. Meanwhile, the consent and support of the students’ supervising teachers were obtained for this survey. After the survey, the researcher conducted group interviews with the students and found that no students had adverse psychological reactions.

## Author contributions

FM: Formal analysis, Investigation, Methodology, Writing – original draft, Writing – review & editing. CC: Conceptualization, Funding acquisition, Writing – original draft, Writing – review & editing. YX: Formal analysis, Investigation, Methodology, Writing – review & editing. HY: Formal analysis, Investigation, Writing – review & editing. XC: Project administration, Supervision, Writing – review & editing.
